# Left centro-parieto-temporal response to tool–gesture incongruity: an ERP study

**DOI:** 10.1186/s12993-018-0138-7

**Published:** 2018-03-13

**Authors:** Yi-Tzu Chang, Hsiang-Yu Chen, Yuan-Chieh Huang, Wan-Yu Shih, Hsiao-Lung Chan, Ping-Yi Wu, Ling-Fu Meng, Chen-Chi Chen, Ching-I Wang

**Affiliations:** 1 Department of Educational Psychology and Counseling, National Taiwan Normal University, Taipei, Taiwan; 2 Department of Occupational Therapy & Graduate Institute of Behavioral Science, Chang Gung University, Taoyuan, Taiwan; 3 Faculty of Psychology, Technische Universität Dresden, Dresden, Germany; 4 Department of Clinical Psychology, Fu Jen Catholic University, New Taipei, Taiwan; 5 Institute of Neuroscience, National Yang-Ming University, Taipei, Taiwan; 6 Department of Electrical Engineering, Chang Gung University, Taoyuan, Taiwan; 7 Division of Occupational Therapy, Department of Physical Medicine and Rehabilitation, Center for Neural Regeneration, Taipei Veterans General Hospital, Taipei, Taiwan; 8 Division of Occupational Therapy, Department of Rehabilitation, Chiayi Chang Gung Memorial Hospital, Chiayi, Taiwan; 9 Health Center, Taipei Fuhsing Private School, Taipei, Taiwan

**Keywords:** Action semantics, Tool–gesture incongruity, N300, N400, Late negative complex

## Abstract

**Background:**

Action semantics have been investigated in relation to context violation but remain less examined in relation to the meaning of gestures. In the present study, we examined tool–gesture incongruity by event-related potentials (ERPs) and hypothesized that the component N400, a neural index which has been widely used in both linguistic and action semantic congruence, is significant for conditions of incongruence.

**Methods:**

Twenty participants performed a tool–gesture judgment task, in which they were asked to judge whether the tool–gesture pairs were correct or incorrect, for the purpose of conveying functional expression of the tools. Online electroencephalograms and behavioral performances (the accuracy rate and reaction time) were recorded.

**Results:**

The ERP analysis showed a left centro-parieto-temporal N300 effect (220–360 ms) for the correct condition. However, the expected N400 (400–550 ms) could not be differentiated between correct/incorrect conditions. After 700 ms, a prominent late negative complex for the correct condition was also found in the left centro-parieto-temporal area.

**Conclusions:**

The neurophysiological findings indicated that the left centro-parieto-temporal area is the predominant region contributing to neural processing for tool–gesture incongruity in right-handers. The temporal dynamics of tool–gesture incongruity are: (1) firstly enhanced for recognizable tool–gesture using patterns, (2) and require a secondary reanalysis for further examination of the highly complicated visual structures of gestures and tools. The evidence from the tool–gesture incongruity indicated altered brain activities attributable to the N400 in relation to lexical and action semantics. The online interaction between gesture and tool processing provided minimal context violation or anticipation effect, which may explain the missing N400.

## Background

A goal-directed action semantic involves comprehension of the object and its corresponding actions with respect to the context. In previous studies of action semantics, participants were mostly asked to determine the compatibility of the actions in a given situation [1–12]. Presentations of conditions violating action-semantics have often involved orientation or functional mismatch to properly execute the functions of the tools [1, 2], illogical tool substitution (e.g. cutting bread or playing cello with a saw instead of a bread knife or a bow [3–7]), or inappropriate body movements in a given context (e.g., a woman who’s looking at her watch, and carrying a suitcase while walking on a treadmill [4]). Concurrent event-related brain potentials (ERPs) have been analyzed to reveal the neural bases of the cognitive processes of action semantics [1–12]. Although context appears to be an indispensable factor processed with action as a meaningful unit, the gesture itself, which is a fundamental component of a valid action, has largely been ignored in the research field of action semantics. Thus, the present study aims to uncover the neural cognitive processes of tool–gesture incongruity.

### ERPs for action semantics

Since gesture semantics is part of action semantics, a brief review of previous studies on action semantics is provided here. Among previous studies examining action semantics [1–12], the N400 is the most reported neural index revealing the congruency effect of action semantics. On the other hand, some researchers have also reported similar N300/N400 peaking earlier at 300 ms after stimulus onset, which has been assumed to reflect picture-related or action-specific semantic processing [5, 7, 8]. In terms of the N400 component, it was observed at first over the centro-parietal scalp areas in response to word stimuli derived from reading sentences in linguistic paradigms with semantically anomalous endings [13]. Later, similar N400 with more frontal distribution than the linguistic N400 have been reported as the action N400 from non-linguistic materials [1–4, 7, 8]. The N400 is therefore regarded as a neural index which can be elicited across stimuli if they are potentially meaningless and incomprehensible.

### More consideration of the enhanced N400

Several underlying factors can influence the magnitude of the N400 in our consideration. The first is the extent of context violation. During sentence reading tasks, it has been reported that the magnitude of the N400 would be influenced by the cloze probability of a word [13, 14]. For instance, words that complete sentences in a nonsensical fashion (low cloze-probability; e.g., The bill was due at the end of the hour) elicit much larger N400 waves than those semantically appropriate words do (high cloze-probability; e.g., The bill was due at the end of the month) in a text [14]. Comprehension of linguistic and non-linguistic semantics is processed based on broad similarities, thus the neural activity patterns resulting from semantically anomalous information in a linguistic domain may show up along with non-linguistic domains such as action semantics. Second, the structural complexity of the background or peripheral context may also be a determining factor. The more abundant the structure is, the more the visual cues and/or that artifacts are provided, thereby influencing the effect of congruity. Third, whether the stimuli are presented in dynamic-, serial-, or static-, influences the topographical distribution and the magnitude of N300/N400 component [3, 7, 8, 10, 11]. For instance, Wu and Coulson [10] reported reduced N400 amplitude for serial cartoon segments, compared to static-image paradigms. Taken together, the N400 appears to be a high-context-dependent component. In this regard, the present study intends to minimize the peripheral factors such as illogical context violation or redundant background information, thereby manifesting the congruency effect of tool–gesture semantics.

### Gesture semantics in previous ERP researches

Here we further review those studies using gestures as the main stimuli. Gestures, which are central to communication, have been found to trigger the N300 and N400 during the process of discriminating the semantics of hand postures [15]. Shibata et al. [16] used EPRs to evaluate the appropriateness of cooperative actions using pass-and-receive paradigms. Pictorial stimuli were presented in a series: first, a preshaped passing hand (e.g., an object placed at the hollow of the palm), then a receiving hand (e.g., palm down as the appropriate receiving action, palm up as inappropriate one), followed by a blank interval. It was found that an inappropriate receiving action elicited a more widely distributed cortical response than did an appropriate action, and the maximum N400 was located in the parietal region. The parietal N400, which is different from the fronto-central N400 reported for the context-violation paradigm, was thought to be semantics processing related to the prediction of interpersonal actions between two people.

Bach et al. [1] further investigated the appropriateness of tool-use actions by classifying mismatch conditions into “functional mismatch,” which involves instruments paired with normally inappropriate target objects (e.g., screwdriver to keyhole) and “orientation mismatch,” which relates to inconsistent spatial properties between the motor action and the target (e.g., orthogonal orientation between insertion and slot). The results of the varied latency of N400 indicated that action and object semantics derive from different sub-processes related to functional and orientation domains, respectively. In line with Bach et al. [1], Balconi and Caldiroli [2] reported a topographical difference in object-related action comprehension, where the significant N400 was observed in the fronto-central area for incorrect object use and predominantly in the temporo-parietal area for unusual object use.

More currently, Proverbio et al. [17, 18] proposed a left hemispheric asymmetry in the activation of premotor and somatosensory areas involved in object perception, which was associated with tool manipulability. They further used the ERPs to examine the neural responses to the visual presentation pictures depicting unimanual (e.g., a hammer) and bimanual (e.g., a handlebar) tools [19]. In the time window of 230–260 ms, the N2 amplitude was elicited at the left parietal cortex, followed by N400 (350–450 ms) at the right parietal cortex. Regardless of the time series, both components were found to be activated in the left premotor cortex. Specifically, only unimanual tools were related to the activation of the left postcentral gyrus in the second time window. This pattern of results suggests a role of the left hemisphere in the neural representation of grasping in right handed people, especially for the N2 component.

Though electrophysiological responses to appropriateness between action and tool have been assessed, the paradigms were quite divergent, hence, less consistent inferences could be concluded. Further, no straightforward evidence up to present has been proposed for understanding the compatibility of tools and manipulation of hand gestures. The present study would be the first to report brain activities involved in tool–gesture congruency, using the tool–gesture paradigm without the confounding factor of context violation and the effect of anticipation.

### Late waveforms beyond N400

In addition to the N400 component, a late positive complex (LPC) after N400 has been observed in some recent studies [1, 5, 10, 12], while late negativity has been found in others [7, 8, 16]. Regardless of its polarities, researchers have assumed this late effect as a reevaluation of the available knowledge of goal-related requirements related to real-world actions [5] or decision-making-related processes [10]. The continued late effect suggests that N400 is not the final stage of the semantic process [14, 20]. Using EPRs enables us to investigate tool–gesture semantics in good time domain analysis, and whether later effect of tool–gesture compatibility occurs after 400 ms can therefore be determined.

In sum, the primary goal of this study is to investigate tool–gesture incongruity using an intra-gesture experimental design with the ERP technique. Based on previous literature, we preliminarily hypothesize that incorrect tool–gesture pairs elicit greater negative N400 amplitude than correct tool–gesture pairs do. Furthermore, a late waveform is expected because the task is relatively difficult and requires a greater degree of visual and cognitive deconstruction than those in previous studies. By means of the ERP recordings, the present study should reveal tool–gesture semantics processing with respect to tool manipulation.

## Methods

### Participants

Twenty healthy university students (nine male) aged 18–24 years (mean = 20.25 years, SD = 1.55), all of whom are right-handed, were recruited in this study (laterality quotient = 83.00 ± 18.66 for handedness based on the Edinburgh Handedness Inventory) [21]. Inclusion criteria for all participants included normal or corrected-to-normal vision. Participants who had suffered from neurologic diseases, hospitalized, under medication, consumed alcohol or tobacco within 6 weeks prior to the experiment were excluded.

The present study recruited healthy adults participating according to their free will via the Internet and was carried out with a non-invasive method. All participants were provided with verbal and written instructions of all the details of the experiment. After understanding and providing consent to participate in this study, personal information was then provided and the questionnaires used in this study were filled in thereafter. All participants could withdraw at any time. The participants did not expect to obtain any benefit from being in this research study. Each participant received a reasonable fee as compensation for their inconvenience and commute expenses.

### Stimuli

**Fig. 1:**
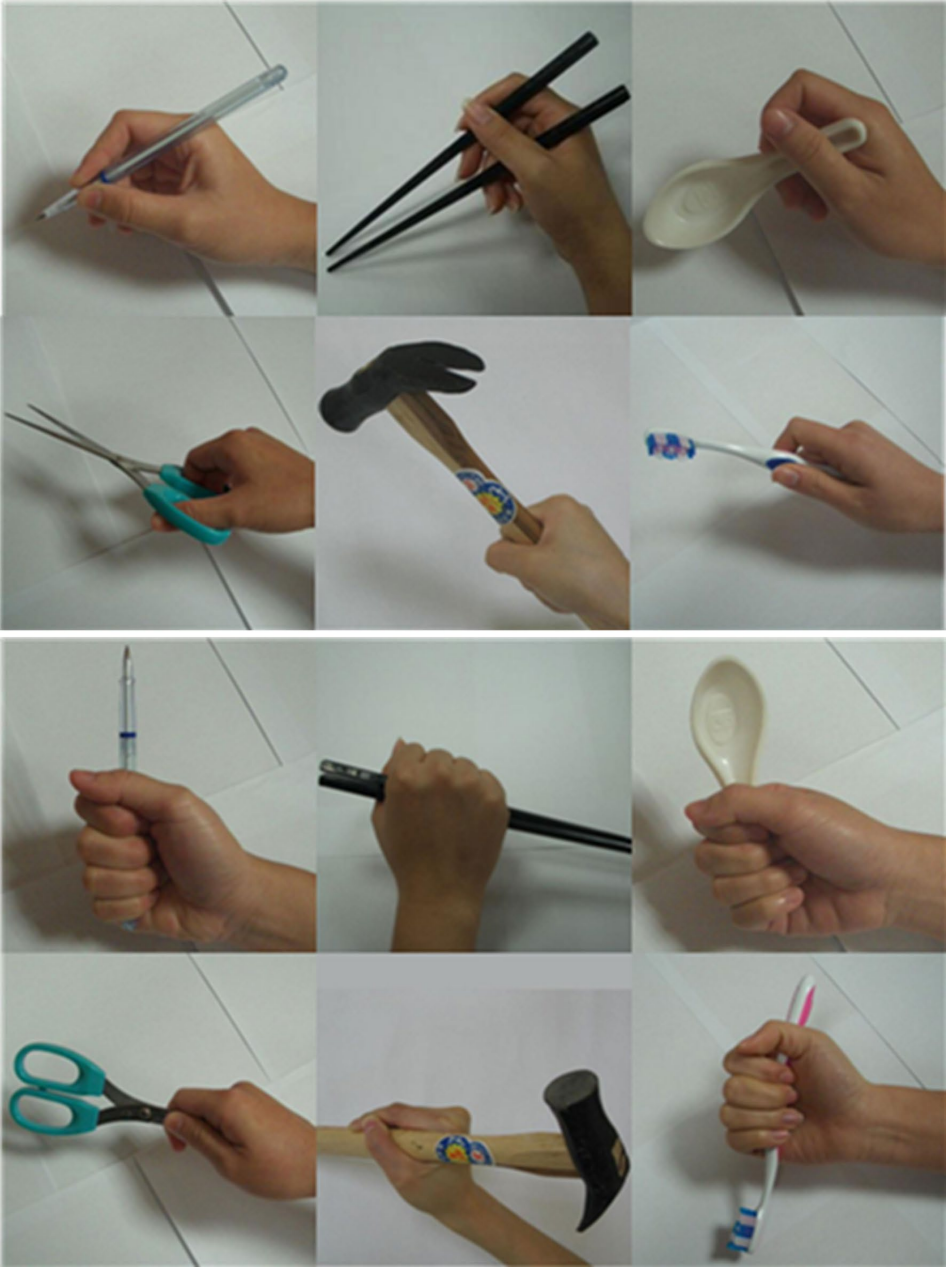
Examples of congruent (upper part) and incongruent (lower part) tool–gesture pairs used in this study

Six commonly used tools, including a pair of chopsticks, a pair of scissors, a pen, a hammer, a toothbrush, and a spoon, were used as the stimuli (Fig. 1). Each frame combined a hand gesture and one of the tools together as a unit. In correct conditions, the tool was manipulated by a gesture that enabled its functional use, whereas in incorrect conditions, the tool was manipulated by an unusual or incomprehensible hand gesture that lacked a goal-directed function. To be more precise, participants were shown images of tools being manipulated with (1). correct gestures: a pen, toothbrush, chopstick, and spoon held in a tripod grasp, a hammer held in a power grasp, and a pair of scissors held with the thumb in the front hole as mover, the index and middle finger in the back hole as stabilizer (Fig. 1 upper section), or (2). incorrect gestures: a pen, toothbrush, chopstick, and spoon held in a tight fist or hook grasp, a hammer and a pair of scissors held in a disoriented manner that disabled the tool’s normal functions (Fig. 1 shows the lower section). Each frame was repeated 20 times. A total of 240 trials composed of tools with correct gestures (120 trials) and incorrect gestures (120 trials) were presented to the participants.

We also invited another 24 college students to judge whether each frame was a correct or incorrect pair. Accuracy was 95% for the correct condition and 89% for the incorrect condition (for more detail please see Appendix). Based on previous studies, we choose static images to minimize the effect of anticipation which may result from sequential presentation. All stimuli were photographed with a 3-megapixel digital camera and edited with Microsoft Window’s built-in Paint software. The stimuli were 15 cm × 15 cm high-resolution photos presented in random order at the center of a 17-inch, 1024 × 768 pixel desktop computer screen. The stimuli were modified referring to the images proposed by Wu et al. (Fig. 1) [22]. Moreover, besides the gesture and the tool themselves, any other visual hints from the background were removed. With the high commonality of pictorial structure between each stimulus, the core neural activities indicating tool–gestures semantics should be revealed. This experimental design enabled us to test whether gestures can affect comprehension in the absence of other sources of semantic input and assess how gestures undergo action semantic processing.

### Procedure

Participants were seated in a dimly lit room approximately 1 m from the computer monitor used for stimuli presentation, and they were fitted with a 32-electrode cap. Each participant was asked to complete 2 blocks of tool–gesture judgment tasks using the contrary respond mode. Each frame was presented for 300 ms. A time window of 1700 ms was allowed for valid responses after stimulus onset. The inter-trial interval (ITI) was 2000 ms. Six additional trials were used in a practice block.

During the first block, participants were asked to judge the tool–gesture pair by pressing button ‘1’ with the index finger for “correct” and button ‘2’ with the middle finger for “incorrect” using their right hand. To counterbalance behavioral response, contrary response mode was used in the second block (by asking participants to press button ‘2’ with their middle fingers for “correct” and ‘1’ with the index finger for “incorrect” using their right hand). Approximately 3–4 min were needed to accomplish the experiment for each block. Short pauses were allowed between blocks to avoid visual fatigue or other factors that could contaminate the EEG recording. During the experiment, a black background was maintained to avoid background variability. The procedure was modified referring to the previous study by Wu et al. [22].

### EEG acquisition and analysis

The EEG was recorded by BrainVision Recorder (version 1.10; Brain Products, Germany) from 32 electrodes distributed based on the 10–20 system (including electrodes from F3/F4, F7/F8, FC1/FC2, FC5/FC6, C3/C4, T7/T8, TP9/TP10, CP1/CP2, CP5/CP6, P3/P4, P7/P8, O1/O2, FPZ, FZ, FCZ, CZ, PZ, and OZ). Eye artifacts were monitored with four EOG electrodes: two located at the outer canthi of the right and left eyes, and two above and below the center of the right eye. The impedances were kept below 10 kΩ. During recording, all electrodes were referenced to the FCz electrode. The EEG was continuously sampled at 1000 Hz with a band pass filter of 0.01–70 Hz and stored for off-line analysis. Resolution of the amplifier was 0.1 μV.

The offline analysis was then conducted using the BrainVision Analyzer software (version 1.05; Brain Products, Germany). Epoches were started at 200 ms and continued to 1000 ms, following stimulus-locked rules. Incorrect behavioral responses and reactions beyond 1700 ms after stimulus onset were eliminated manually. At least 100 trials were maintained for each condition. Averages were aligned to a 200 ms pre-stimulus baseline. The band pass filter was 0.1–30 Hz (12 dB/octave). Offline data were re-referenced to the average of the TP9 and TP10 electrodes. Segments contaminated by artifacts as amplitudes exceeding ± 100 μV at 4 EOG electrodes, and ± 60 μV at the resting electrodes were detected and excluded for the following analysis. Prior to averaging, ocular artifacts were also corrected by independent component analysis (ICA). Bipolar vertical EOG (VEOG) and horizontal EOG (HEOG) channels were calculated as the difference between VEOU/VEOD and HEOL/HEOR electrodes, respectively. Thirty channels including VEOG and HEOG, and excluding VEOU, VEOD, HEOL and HEOR, were entered into the ICA analysis, resulting in 30 independent components. The components resembling blinks and eye movements were blocked through the inspection of topographic maps and the time course with the EOG channels. Segments were averaged separately for each congruent or incongruent condition.

After group averaging from data of all 20 participants, mean amplitudes for 3 visually prominent components were calculated by self-programmed MATLAB (Mathworks, USA), including N300 (220–360 ms), N400 (400–550 ms) and the late negative complex (LNC, 720–800 ms). The focus of the analyses was the mean amplitudes of these three components in different regions. Twenty-one electrodes were classified into 7 regions for further analysis, including the left-fronto-central area (F7, F3, FC5), left-centro-parieto-temporal area (T7, C3, CP5), left-parieto-occipital area (P3, P7, O1), midline (Fz, Cz, Pz), right-fronto-central area (F8, F4, FC6), right-centro-parieto-temporal area (T8, C4, CP6), and right-parieto-occipital area (P4, P8, O2).

### Statistical analysis

For behavioral data and electrophysiological data, differences were assessed using paired sample *t* tests to correctness (correct/incorrect) as the factor. We further report the effect size of Cohen’s d value to reveal the strength of the relationship between conditions [23]. Statistical differences achieved significance when *p* < 0.05 (two-tailed). p values were adjusted for multiple testing with the Hochberg method for electrophysiological data.

## Results

### Behavioral results

In general, participants responded accurately when deciding the correctness of the tool–gesture stimuli. The participants demonstrated a higher accuracy rate (AR) and faster reaction time (RT) for the correct condition. The mean AR and RT were 96.08 ± 2.90% and 678.33 ± 104.31 ms for correct tool–gesture pairs, and 93.42 ± 4.50% and 701.60 ± 92.42 ms for incorrect tool–gesture pairs, respectively. The differences in both AR and RT between correct and incorrect conditions reached a significant level [t(19) = 2.791, p = 0.012, ES(d) = 0.70 for AR; t(19) = − 3.251, p = 0.004, ES(d) = 0.24 for RT].

### Event-related potentials results

**Fig. 2:**
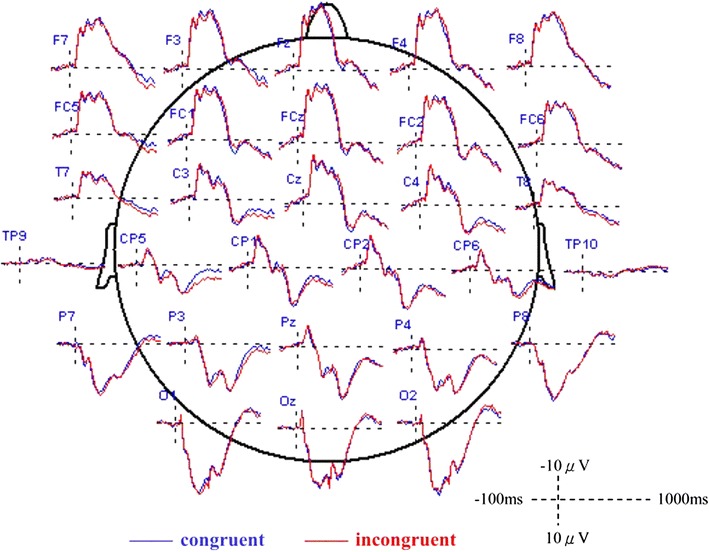
Grand averaged ERPs for correct (blue) and incorrect (red) tool–gesture stimuli

Figure 2 shows the averaged ERP waveforms for correct and incorrect tool–gesture stimuli. In the time period of 220-360 ms after stimulus appearance, correct pictures (mean = − 1.54 µV, SD = 2.06) elicited more negative N300 than incorrect pictures (mean = − 1.03 µV, SD = 2.02) did in the left centro-parieto-temporal area [t(19) = − 2.687, Hochberg adjusted *p* = 0.015, ES(d) = 0.60]. The result indicates the left centro-parieto-temporal N300 was elicited when participants saw comprehensible tool–gesture pairs. None of the other areas showed significant N300 components (all Hochberg adjusted *p* > 0.05). Contrary to our expectations, none of the areas demonstrated significant congruency effects for N400 (all Hochberg adjusted *p* > 0.05). From 720 to 800 ms epochs, correct pictures (mean = 1.10 µV, SD = 2.92) evoked a greater late negativity complex than incorrect pictures (mean = 1.87 µV, SD = 2.47) did in the left centro-parieto-temporal area [t(19) = − 1.979, p = 0.024, ES(d) = 0.55)].

## Discussion

This study investigates action semantic processing for tool–gesture incongruity using an intra-gesture experimental design with the ERP technique. Unlike previous studies exploring the action semantics [1–12], the present study deliberately designed and controlled the stimuli to minimize the effect of context violation and anticipation, for the purpose of revealing the core neural activities indicating tool–gesture semantics. According to the present findings, participants were less accurate and approximately 20 ms slower in discerning incorrect tool–gesture pairs. Two main findings were derived from our ERP data: correct tool–gesture pairs elicited (1). more negative N300, and (2). more negative late negativity complex (LNC) than incorrect pairs did in the left centro-parieto-temporal area. However, we did not find the typical N400 component for the semantic processing of linguistic material [13, 24–26] or action-related material [1–4, 11, 16, 20]. Our results imply that the neural mechanism for comprehending tool–gesture semantics is different from those for understanding action semantics with respect to context.

### Raised N300: object recognition of semantic memories

In this study, we found more negative N300 elicited by correct tool–gesture pairs in the left centro-parieto-temporal area; this may represent the neural processes for perceptual object recognition [5, 7, 8, 13, 27, 28] and structural description matching [29, 30]. The present finding was in line with Proverbio et al. [17, 18], who reported left hemispheric asymmetry in the activation of premotor areas involved in object perception in right handers, which was associated with tool manipulability, specifically for gestures. In addition, the anterior temporal cortex has been suggested as a primary semantic source of top-down influences involved in object recognition [31]. In brief, the result from the present study indicates that object recognition, which is mainly addressed by the left centro-parieto-temporal area, is the first stage of neural processing of tool–gesture incongruity in right handers.

Among previous studies investigating action semantics using neurolinguistic paradigms, the enhanced recognition potential (RP, or N250) between 250 and 350 ms with an occipitally distribution has been reported for visually recognized and semantically comprehended actions within the context reference [3, 4]. Consistent with the above findings, the present study found that when the tools were accompanied by a gesture that conveyed the functional meaning (the *correct* condition in this study), the visually recognizable characters lead to an increase in N300 amplitude. Whereas in *incorrect* conditions, the tools were manipulated with an unusual or incomprehensible hand gesture, which lowered the possibility for recognizing the congruence between tool and gesture in visual processing, hence resulting in decreased N300.

In this study, the effects resulting from contextual violations and incongruent contexts were minimized. Thus, the participants therefore had to pay more attention to the structural matching between the tools and the gestures to make the right judgment. Buxbaum et al.’s study [32] inferred that the hand shaping for object use additionally requires access to stored knowledge about the skilled manipulation specific to a given object. Note this inference is in accordance with the reported finding of the observed enhanced N300 when semantic expectations matched the baseline. Some authors therefore have suggested the N300 as an index in the rapid matching of visual input to stored semantic knowledge [13, 27, 33]. This idea was also supported by an object and action identification study, wherein an increased N3 complex was observed for successful category decisions with intact known objects rather than scrambled ones [34]. Therefore, an increased N300 effect for correct tool–gestures is consistent with the findings for object identification tasks, which suggests that N300 is involved in perceptual object categorization of visual stimuli based on semantic memories.

### Reasons for the lack of N400

Since N400 has been considered as a strong neural index across linguistic and action semantic researches [1–14], we were looking forward to verifying the role of N400 as a representative component initially. In light of action semantics, Reid and Striano [11] interpreted the N400 response as the rapid indexing of neural system activities, discerning semantic information in actions, and anticipating information within goal-directed actions [14]. Based on our experimental design, however, insignificant N400 waves between conditions were reported. According to the present findings, we inferred that understanding the way gestures undergo action semantic processing with tools is different from understanding action semantics with context violation. We suggest it is the substantially structural similarities, the subtle distinctions between the correct and incorrect tool–gesture pairs, which may have bothered the participants’ ability to rapidly match the semantic information conceptually, as suggested by Reid and Striano [11], hence resulting in the insignificance of N400 between conditions.

Another possible explanation for the diminished N400 may be related to stimulus repetition and familiarity. Because of the small set of only six (tools), participants could become familiar with how to categorize them easily (as incorrect) after a few repetitions. It is possible that the repetition and familiarity effects had the power of reducing the incongruence effect.

### Enhanced LNC for the secondary reanalysis of tool–gesture incongruity

The present findings indicate that a quick matching of N300 or N400 appears to be insufficient for understanding tool–gesture incongruity. Rather, a LNC should be responsible for the secondary reanalysis of the functional semantics and consistency between gesture and tool. Late deflections in context deviation paradigms in previous studies were interpreted as a reanalysis or reevaluation process, which have mainly been reported with broad neural activities across the frontal to parietal lobes [5–7, 12, 24]. The time-serial result was in line with the notion that brain dynamics for tool–gesture semantic processing is similar to that of lexical semantics in the later stage; however, the dominant area for the secondary reanalysis of tool–gesture incongruity has mainly been addressed by the left centro-parieto-temporal area [3, 7, 8, 11].

Late negative complex (LNC) has been investigated in memory studies and found to reflect processes that are engaged in the tasks of color source retrieval [35], when task-relevant memory features require more evaluation [36], and in action monitoring and contextual retrieval [37]. In this study, functional construction of tool–gesture stimuli is considered to be formed by previous knowledge rather than anticipation. Hence, the online interaction between gesture and tool may be inclined toward the memory retrieval process rather than prediction. The increased late effect indexes the efforts when participants reintegrate the information of the tool and the manipulated hand gesture as a whole. This retrieval process helps to build meaning by mental spatial manipulation based on users’ prior experience and object knowledge.

The LNC contributes particular meaning, apart from that reported in previous studies, to the tool–gesture stimuli. We propose that discriminating between the incorrect and the correct tool–gesture pair in such a high similarity condition is relatively mind-consuming for visual perception, thus a visually-dependent secondary reanalysis of the functional semantics and consistency between gesture and tool is necessitated. In addition to the perquisite knowledge of tool-identification and ideomotor praxis, it is the resemblance of the pictorial stimuli that prompted a more skilled visual analysis to reevaluate the compatibility and the visual-spatial construction of the tool–gesture pair as a unit. More visual and cognitive loading therefore facilitated the left centro-parieto-temporal neural activities during gesture-semantic judgment.

## Limitations

Several limitations and underlying factors may have influenced the outcomes and the inferences of the present study. First, the stimulus set was small (only six tools with twelve stimuli were used). The more times each visual stimulus was repeated, the high familiarity of the incorrect pairs might have decreased such difficulty in categorization/recognition. Second, in an object-related action comprehension study, Balconi and Caldiroli [2] found N400-like event-related potentials with different topographical distributions for “unusual” or “incorrect” object use. However, this study provided the dichotomous decision of “correct” or “incorrect” rather than subdividing these visual stimuli into more detailed categories, as in Balconi and Caldiroli’s research. A more detailed categorization may help future studies of brain topography related to action semantics.

Third, because a gesture is a component comprised of complex dual orientation and function meanings, it is difficult to discriminate function mismatches from orientation mismatches such as those studied in Bach et al. [1]. Fourth, could presentation of hand gesture itself (e.g., a picture showing only tripod grasp or power grasp without tools) elicit differential brain activities for visual analysis? The findings from an additional control experiment (unpublished data) demonstrated that within each time window of interest, neural activities for only a hand gesture without tool presentation were insignificant between correct and incorrect conditions. The result from the control experiment helped us to rule out the confounding possibility of the gesture itself. Fifth, longer reaction times and lower accuracy rates indicate the difficulty of judging incorrect tool–gesture pairs. The difficulty of the task itself may have strengthened the anticipated results. Rigorous experimental designs are needed for future studies. Lastly, the present findings demonstrating the left-hemispheric dominance of tool–gesture incongruity may be generalized to right-handers only. Using the transcranial magnetic stimulation (TMS)-induced motor evoked potentials (MEPs) technique, Sartori et al. [38] reported that regardless of the laterality of the hand being observed, the motor resonance is noted in the observer’s dominant effector for both left- and right-handers. Due to the concern about effector-independent motor representations, the current finding of left-hemispheric dominance of tool–gesture incongruity might be reversed in left-handers. Recruiting left-handers would help further clarify such issue.

## Conclusion

This study focused on tool–gesture action semantics congruency. Our study showed conclusively that the left centro-parieto-temporal area was the dominant brain region contributing to the neural processing of tool–gesture action semantics in right handers. The temporal brain dynamics indicate that the N300 was evoked and indexed as the neural processing of object recognition based on semantic memory in the first stage. Later, a late negative complex (LNC) was evoked and indexed as the visually-dependent memory retrieval process for the secondary reevaluation of tool–gesture compatibility. Unlike previous studies reporting consistent N400 across the investigation of linguistic or action semantics [1–14], the tool–gesture paradigm from the present study reports no N400 response. The reason for the lack of N400 may be related to the absence of context violation, the effect of anticipation, and the high similarities of the visual-spatial constructions of the stimuli used in this study. The specific relationship between the activated cortical area and types of linguistic and/or action semantic violations merit further discussion.

## Appendix

See Table 1.

**Table 1:** Accuracy of the judgment of tool–gesture pairs (N = 24)

Categorization	Accuracy	
	Correct	Incorrect
Correct		
Chopsticks	20/24	4/24
Scissors	24/24	0/24
Hammer	23/24	1/24
Spoon	22/24	2/24
Pen	24/24	0/24
Toothbrush	24/24	0/24
Incorrect		
Chopsticks	1/24	23/24
Scissors	12/24	12/24
Hammer	1/24	23/24
Spoon	2/24	22/24
Pen	0/24	24/24
Toothbrush	0/24	24/24
